# Supplementary material for: Formation of calcium sulfate through the aggregation of sub-3 nanometre primary species
Source: Nat Commun. 2016 Apr 1;7:11177. doi: 10.1038/ncomms11177 (PMC4821993; doi:10.1038/ncomms11177)
Supplement: Supplementary Information — Supplementary Figures 1-7, Supplementary Tables 1-2, Supplementary Equations 1-9, Supplementary Notes 1-9 and Supplementary References. [file ncomms11177-s1.pdf]

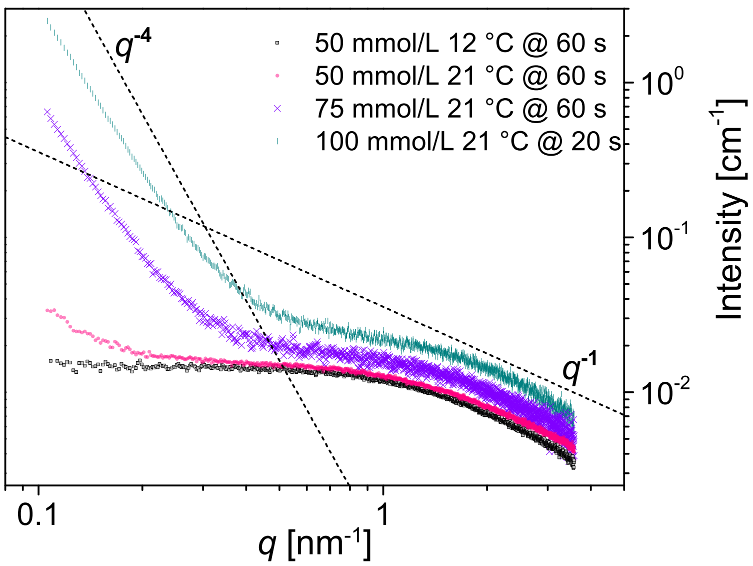

Supplementary Figure 1. Comparison of selected *in situ* SAXS patterns. The patterns were collected at various physicochemical conditions (see legend); Shown is also the change in the $I(q)$ dependence of the scattering exponent to emphasize the invariant in the high-q part of the data (dashed lines) indicating that at $q > 1$ nm^{-1} the pattern originates from identical scatterers. The differences in intensity at $q > 1$ nm^{-1} are attributable to different volume fractions of the primary scatterers forming at different supersaturations (i.e., at higher supersaturations more scatterers exist in the solution).

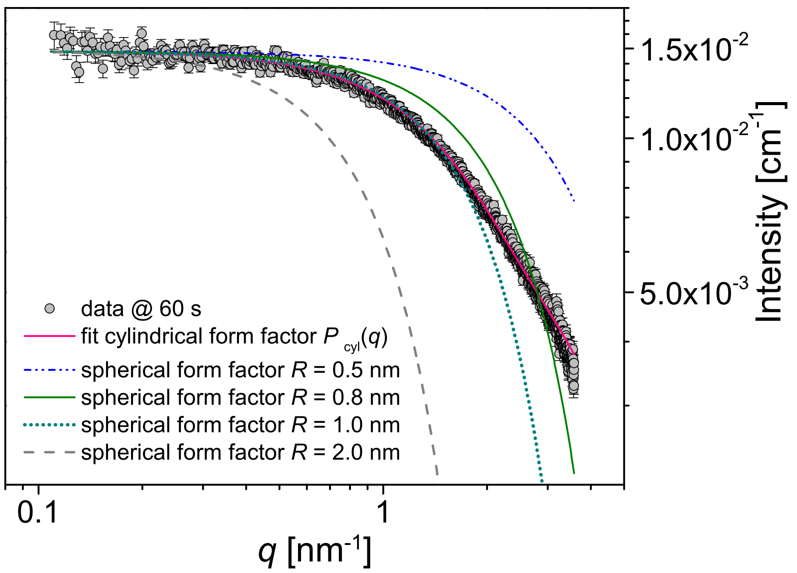

Supplementary Figure 2. Cylindrical form factor. Fit to the data at 60 seconds compared to spherical form factors of various radii values.

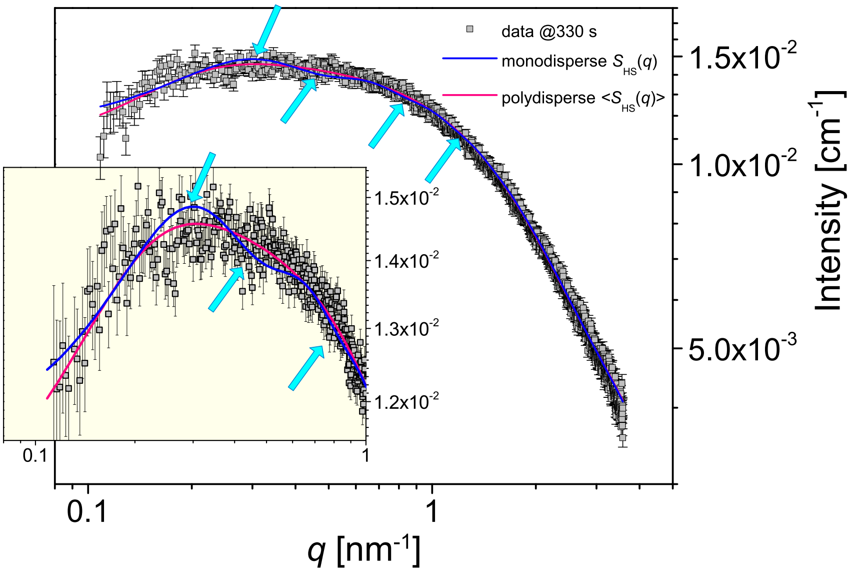

Supplementary Figure 3. Comparison of the effect of the mono- and polydisperse structure factors. SAXS pattern at 330 seconds together with fits (solid lines) in which $P_{cyl}(q)$ is expressed by Supplementary Equation 1 (see Supplementary Note 2) and two expression for the structure factor are compared: monodisperse $S_{HS}(q)$ (blue line, Supplementary Equation 2, Supplementary Note 3), and polydisperse $< S_{HS}(q)>$ (red, Supplementary Equation 4, Supplementary Note 3). Arrows mark the oscillations in the fitted curve caused by the monodisperse $S_{HS}(q)$. The inset shows a part of the scattering pattern relevant for the structure factor.

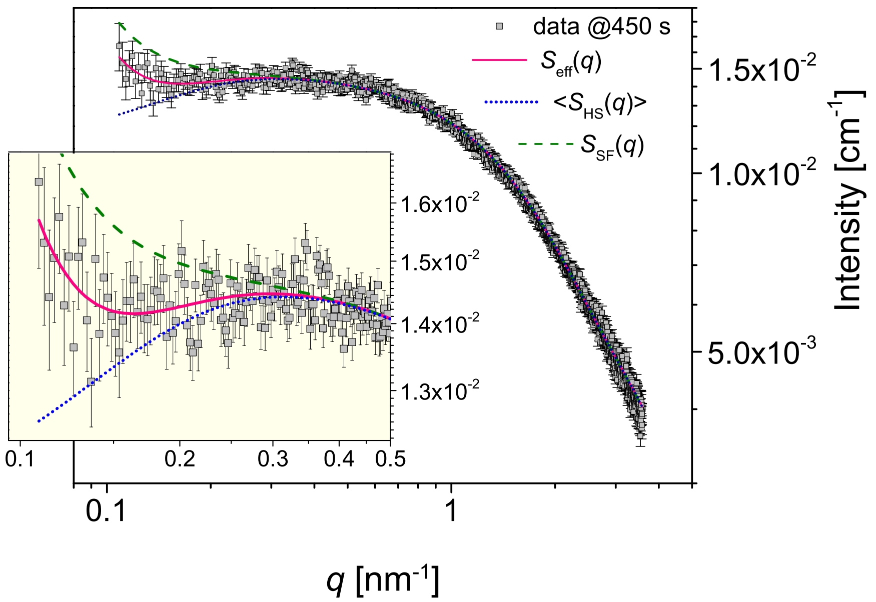

Supplementary Figure 4. Structure factor during the transition between stages II and III. SAXS pattern at 450 seconds together with a fit (red solid line) in which $P_{cyl}(q)$ is a from factor of a cylinder, and the structure factor $S_{eff}(q)$ is expressed as a linear combination of $< S_{HS}(q)>$ and $A'{\cdot}SF(q)$ (see Supplementary Notes 2, 3, 5 & 6). The green dashed and blue dotted lines represent fits of the corresponding expressions considering $<S_{HS}(q)>$ and $S_{SF}(q)$ respectively. The inset shows the detail of the scattering pattern relevant for the structure factor.

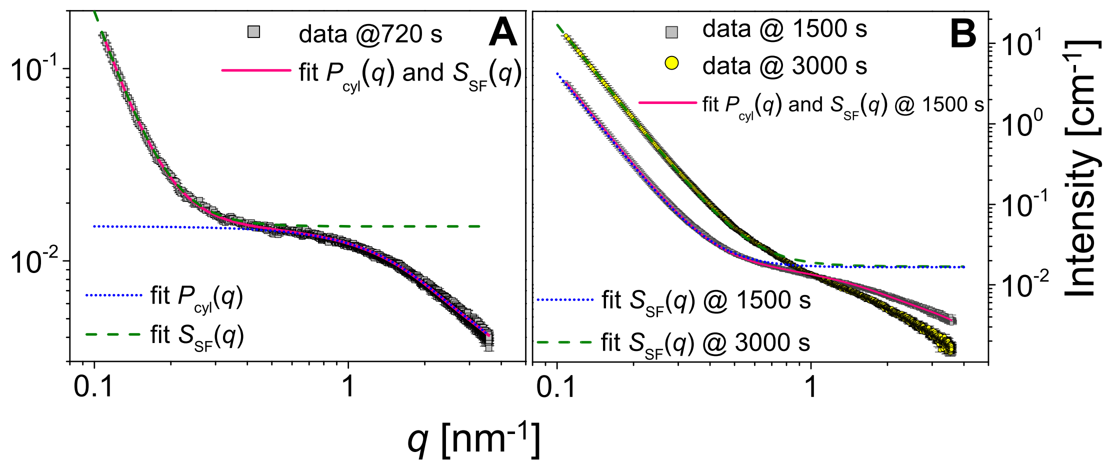

Supplementary Figure 5. Surface fractal structure factor. SAXS patterns obtained from the experiment with a 50 mmol/L $CaSO_4$ solution reacted at 12 °C showing different parts of the reaction. A) Intensity change in the SAXS pattern collected at 720 seconds, and B) at 1500 and 3000 seconds; various fits are also shown (dashed and solid lines according to the inset legends).

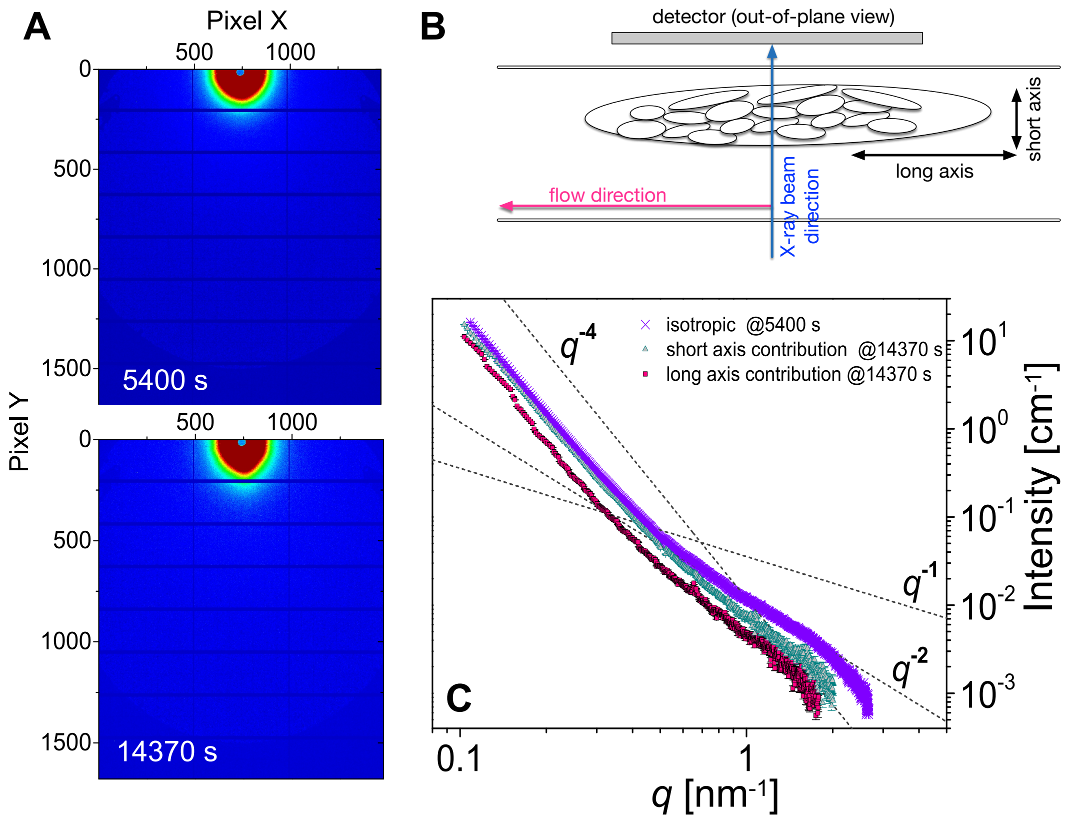

Supplementary Figure 6. Scattering after 5400 seconds. A) 2D SAXS patterns from $CaSO_4$ 50 mmol/L at 12 °C at 5400 and 14370 seconds; B) Schematic representation of the morphology of flow-oriented particles; C) 1D SAXS curves for the same conditions. Shown also is the change in the $I(q)$ dependence of the scattering exponent to emphasize the differences in the high-q part of the data (dashed lines).

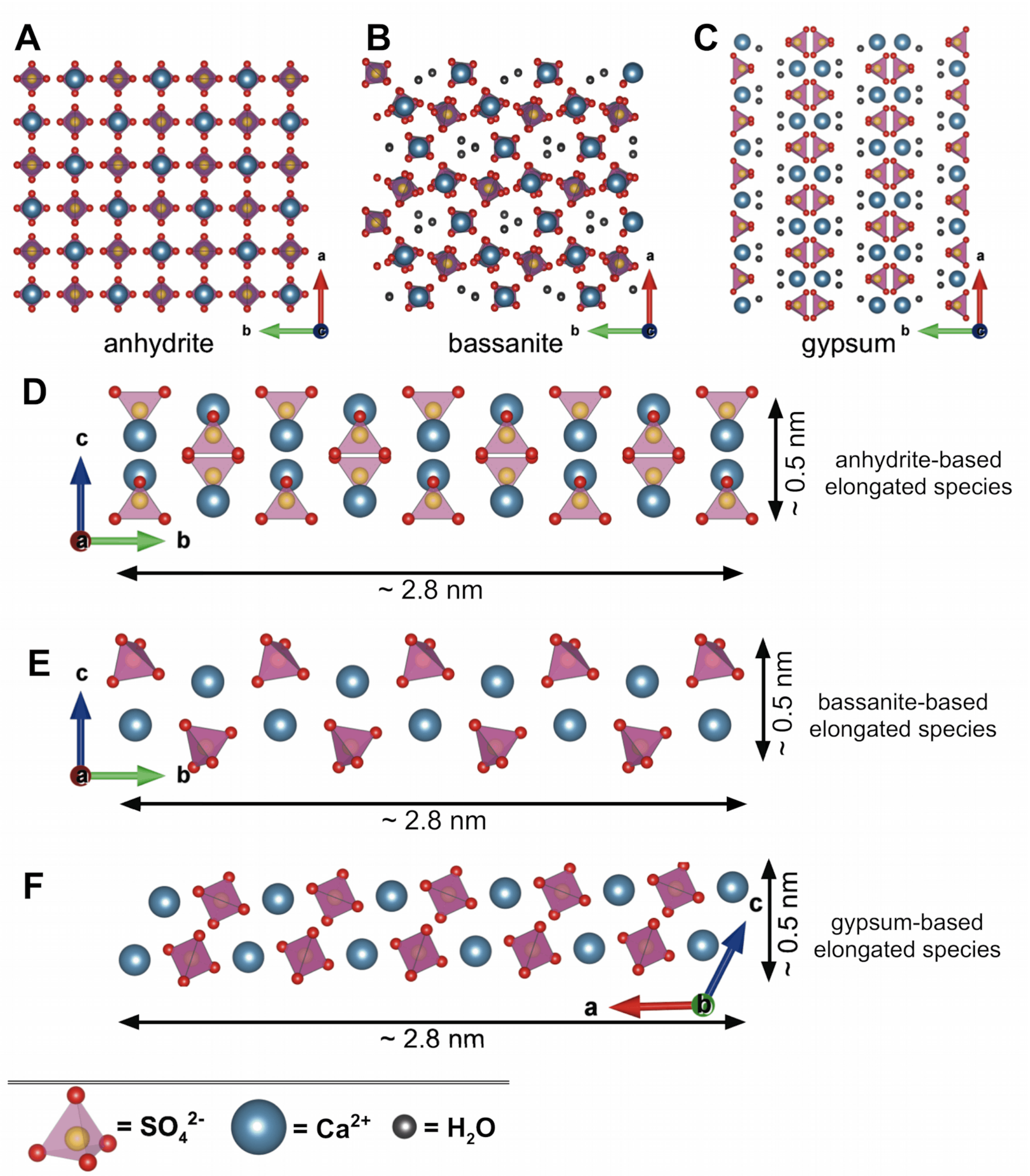

Supplementary Figure 7. Visualisations of $CaSO_4$ phases. Projections[1] along *c*-axis for A) $CaSO_4$, anhydrite[2] (AMCSD 0005117); B) $CaSO_4 \cdot 0.5H_2O$, bassanite[3] (AMCSD 0006909); C) $CaSO_4 \cdot 2H_2O$, gypsum[4] (COD 2300259); Proposed structures of $CaSO_4$ elongated primary species based on D) anhydrite; E) bassanite; F) gypsum.

Supplementary Table 1. Overview of physicochemical conditions and $CaSO_4$ development stages. The WAXS time-periods correspond to the induction times at which the intensity of the (020) diffraction peak of gypsum was above background. Note the change in the time length/frame between experiments; these time frames and total experimental lengths were chosen based on previous work[5, 6, 7] and match the increase in reactions rates at the higher temperatures and higher supersaturations.

	**50**<br>[$CaSO_4$] [mmol/L]	**75**<br>[$CaSO_4$] [mmol/L]	**100**<br>[$CaSO_4$] [mmol/L]	**150**<br>[$CaSO_4$] [mmol/L]
**12 °C**	30 seconds *per* frame,<br>480 frames<br>SAXS: stages I-IV<br>WAXS: ~1500 seconds			
**21 °C**	30 seconds *per* frame,<br>400 frames<br>SAXS: stages III-IV<br>WAXS: ~750 seconds	1 seconds *per* frame,<br>600 frames<br>SAXS: stages III-IV<br>WAXS: ~140 seconds	1 second *per* frame,<br>600 frames<br>SAXS: stages III-IV<br>WAXS: ~60 seconds	1 second *per* frame,<br>600 frames<br>SAXS: stages III-IV<br>WAXS: ~40 seconds
**30 °C**	15 seconds *per* frame,<br>500 frames<br>SAXS: stages II-IV<br>WAXS: ~650 seconds			
**40 °C**	15 seconds *per* frame,<br>240 frames<br>SAXS: stages II-IV<br>WAXS: ~400 seconds			

Supplementary Table 2. Values of molar mass, density, electron density and solubilities for the three polymorphs at 12 °C.

Phase	Molar mass [g/mol]	Density [g/cm^3]	Electron density [e$^-$/cm^3]	Solubility in pure H_2O [mmol/L]	Solubility in 100 mmol/L NaCl solution [mmol/L]
$CaSO_4 \cdot 2H_2O$ (gypsum)	172.17	2.31 - 2.33	711 - 717	15.48	22.75
$CaSO_4 \cdot 0.5H_2O$ (bassanite)	145.15	2.69 - 2.76	815 - 836	101.20	111.90
$CaSO_4$ (anhydrite)	136.14	2.97	893	23.13	30.37

Supplementary Equation 1:

$$I(q,R,L,\phi)=\phi V_{\mathrm{part}}(\Delta\rho)^2\cdot P_{\mathrm{cyl}}(q,R,L)\ \rightarrow$$

$$\rightarrow\ P_{\mathrm{cyl}}(q,R,L)=\int_0^{\pi/2}F^2(q,R,L,\alpha)\sin\alpha\ \mathrm{d}\alpha=\int_0^{\pi/2}\left[\frac{2J_1(qR\sin\alpha)}{qR\sin\alpha}\cdot\frac{\sin(\frac{1}{2}qL\cos\alpha)}{\frac{1}{2}qL\cos\alpha}\right]^2\sin\alpha\ \mathrm{d}\alpha$$

Supplementary Equation 2:

$$S_{\mathrm{HS}}(q,v,R_{\mathrm{eHS}})=\frac{1}{1+24v\left\{G(q,v,R_{\mathrm{eHS}})/(2qR_{\mathrm{eHS}})\right\}} \qquad \text{(a)}$$

The function $G(q,v,R_{\mathrm{eHS}})$ has the following form:

$$G(q,v,R_{\mathrm{eHS}})=A\frac{\sin(2qR_{\mathrm{eHS}})-(2qR_{\mathrm{eHS}})\cos(2qR_{\mathrm{eHS}})}{(2qR_{\mathrm{eHS}})^2}+B\frac{2(2qR_{\mathrm{eHS}})\sin(2qR_{\mathrm{eHS}})+\left[2-(2qR_{\mathrm{eHS}})^2\right]\cos(2qR_{\mathrm{eHS}})-2}{(2qR_{\mathrm{eHS}})^3}+$$
$$+C\frac{-(2qR_{\mathrm{eHS}})^4\cos(2qR_{\mathrm{eHS}})+4\left[3(2qR_{\mathrm{eHS}})^2-6\right]\cos(2qR_{\mathrm{eHS}})+4\left[(2qR_{\mathrm{eHS}})^3-6(2qR_{\mathrm{eHS}})\right]si(2qR_{\mathrm{eHS}})+24}{(2qR_{\mathrm{eHS}})^5} \qquad \text{(b)}$$

, where A, B and C are dependent on the local volume fraction, v, through the following expressions:

$$A=\frac{(1+2v)^2}{(1-v)^4},\quad B=\frac{-6v(1+v/2)^2}{(1-v)^4},\quad C=vA/2 \qquad \text{(c)}$$

Supplementary Equation 3:

$$R_{\mathrm{g}}=\sqrt{\frac{R^2}{2}+\frac{L^2}{12}}$$

Supplementary Equation 4:

$$<S_{\mathrm{HS}}\left(q,v,<R_{\mathrm{eHS}}>,\sigma\right)>\ =\ \frac{\int_0^{\infty}D\left(<R_{\mathrm{eHS}}>,\sigma,r\right)\cdot S_{\mathrm{HS}}\left(q,v,r\right)\mathrm{d}r}{\int_0^{\infty}D\left(<R_{\mathrm{eHS}}>,\sigma,r\right)\mathrm{d}r}$$

Supplementary Equation 5:

$$D\left(< R_{\mathrm{eHS}} >, z, r\right) = \frac{r^{z}}{\Gamma(z+1)}\left(\frac{z+1}{< R_{\mathrm{eHS}} >}\right)^{z+1} \exp\left(-\frac{z+1}{< R_{\mathrm{eHS}} >} r\right)$$

Supplementary Equation 6:

$$\sigma = \frac{< R_{\mathrm{eHS}} >}{\sqrt{z+1}}$$

Supplementary Equation 7:

$$I_{SF}(q, D_{\mathrm{s}}, A) = A \cdot SF(q, D_{\mathrm{s}}) \qquad \text{(a)}$$

$$SF(q, D_{\mathrm{s}}) = \frac{\Gamma(5 - D_{\mathrm{s}}) \sin[\pi(3 - D_{\mathrm{s}})/2]}{3 - D_{\mathrm{s}}} q^{-6+D_{\mathrm{s}}} \qquad \text{(b)}$$

Supplementary Equation 8:

$$S_{SF}(q, D_{\mathrm{s}}, A') = 1 + A' \cdot SF(q, D_{\mathrm{s}})$$

Supplementary Equation 9:

$$S_{eff}(q) = < S_{\mathrm{HS}}(q, < R_{\mathrm{eHS}} >, z) > + A' \cdot SF(q, D_{\mathrm{s}})$$

Supplementary Note 1:

Evolution of the scattering patterns at various physicochemical conditions

We investigated the formation of solid $CaSO_4$ phases in various supersaturated solutions with concentrations up to 150 mmol/L and at temperatures between 12 and 40 °C. In all cases changes in SAXS and WAXS intensities and shapes of the patterns were observed over time and these changes were correlated with supersaturation levels and temperature (see Supplementary Table 1). The analysis of the time-resolved SAXS patterns indicated that the scattering features for all experiments evolved in an equivalent manner, regardless of solution conditions. Thus, for simplicity, we discussed in the main text in detail the structural information contained within the scattering patterns based on the 50 mmol/L $CaSO_4$ experiments measured at 12 °C. For the experiments at higher supersaturations (75 – 150 mmol/L) and higher temperatures (21, 30 and 40 °C), the higher reaction rate[5] prevented us in some cases to clearly distinguish all 4 stages (Supplementary Table 1). Nevertheless, the start of the reaction in the experiment at the lowest supersaturation (12 °C and 50 mmol/L; Supplementary Fig. 1) can be compared with selected patterns at similar time frames from experiments at a higher temperature (21 °C) and higher supersaturations (50 – 100 mmol/L; Supplementary Fig. 1), revealing differences in the early stages. Each curve shows different characteristic intensities at $q < 1$ nm^{-1} (increase with concentration and temperature) indicating that at comparable reaction times the structures at 21 °C and higher supersaturations already reached a more advanced stage of development. The low-q profiles resemble those of stage III for the 50 mmol/L, 12 ºC experiment (see the main text, Fig. 1A). Important to note, is that at $q > 1$ nm^{-1} all scattering curves have nearly identical profiles (Supplementary Fig. 1),

clearly demonstrating that, despite the differences in solution supersaturation, the primary species are equivalent in terms of shape and size.

Supplementary Note 2:

Mathematical expressions for scattering from cylindrical species

The first four scattering curves in Fig. 2A in the main text (30 – 120 seconds) contain a clear Guinier region[8, 9] *i.e.,* a plateau at $I(q) \propto q^0$ for $q < 1$ nm^{-1}, and a region for which $I(q) \propto q^{-1}$ for *ca.* 1.5 nm$^{-1} > q > 3.0$ nm^{-1}. The fact that a $I(q) \propto q^0$ dependence is found at low-q for $q \rightarrow 0$ indicates that scattering originated from non-aggregated and non-interacting species. Furthermore, the occurrence of the $I(q) \propto q^{-1}$ dependence at high-q is typically attributable to scattering from elongated, anisotropic shapes[8]. Therefore, for stage I, the scattering curves could be best fitted with Supplementary Equation 1, which includes the analytical expression for the scattering intensity from the collection of non-interacting homogenous cylindrical objects of length L and radius R (see ref. [8]). In Supplementary Equation 1, ϕ is the volume fraction of scatterers, $\Delta\rho$ the scattering length density difference between a scatterer and the matrix (a solvent), V_{part} is the volume of a single particle (scatterer) and $P_{\text{cyl}}(q,R,L)$ is the form factor of the cylinder. Furthermore, $J_1(x)$ is the first-order Bessel function and α is the angle between the long axis of the cylindrical object and the primary beam. In Supplementary Equation 1, the form factor $P_{\text{cyl}}(q,R,L)$ is equal to the square of the amplitude $F^2(q,R,L,\alpha)$ averaged over all angles α, so that $P_{\text{cyl}}(q,R,L) = \left\langle F^2(q,R,L) \right\rangle_\alpha$. Supplementary Figure 2 illustrates the fit of Supplementary Equation 1 to the data (collected at 60 seconds, see also the main text). In this figure we also compare the cylindrical form factor fit with the analogues

fits based on expression for scattering from spherical particles[8] of various radii, and the same pre-factor as the cylinder. One needs to point out that within the measured q-range the part of the form factor contributing to the scattering from the cylindrical cross-section is represented by data points at $q > \sim 3$ nm^{-1}. Hence, a circular shape for the cylindrical cross section is only assumed for the purpose of fitting, and the resulting values of R constitute the upper limit of this parameter.

Supplementary Note 3:

Mathematical expressions for scattering from the domains of primary species

For all scattering profiles shown in Fig. 2A of the main text, at $q > 1$ nm^{-1}, neither the shape of the profiles nor the intensity values of the scattering features changed, but at $q < 0.3$ nm^{-1}, the intensity systematically decreased between 150 and 390 seconds (stage II). We attribute this change to an evolution of the inter-particle structure factor $S(q)$, which resulted from the increase in particle-particle interferences between the primary species in solution. In our analysis we considered a simplified structure factor, $S_{\mathrm{HS}}(q)$, that only accounts for the interactions through the hard-core repulsive potential[10] expressed by Supplementary Equation 2, where $S_{\mathrm{HS}}(q)$ depends on the local volume fraction of interacting neighboring scatterers, v, and the effective hard-sphere radius, R_{eHS}, which represents the typical distance between neighboring primary scatterers. Our initial fitting, including the hard-sphere model described in Supplementary Equation 2, also indicated that lengths, radii, and $\phi V_{\mathrm{part}}(\Delta\rho)^2$ pre-factors (see Supplementary Note 2) of the cylindrical form factor were constant throughout stages I and II. Since the geometry of the formed primary entities remained constant we can assume that their electron density did not change either, and invariably it follows that their volume fraction ϕ also remained constant.

The actual physical dimensions of interacting cylindrical particles can be compared to the typical distance between neighbouring particles, by relating the effective hard-sphere radii, R_{eHS} (from Supplementary Equation 2) with the radii of gyration (R_g) of the particles. The gyration radius, R_g, of a cylinder of length L and radius R is expressed by Supplementary Equation 3. Our data indicate that $R_{eHS} >> R_g$, and this implies that, on average, the distance between the formed primary particles was sufficiently large to prevent aggregation. By taking into account the constant value of $\phi V_{part}(\Delta\rho)^2$, and the fact that R_{eHS} values are large (> 8 nm), we propose that these primary species form domains of locally increased scatterer number densities separated by regions depleted of scatterers, *i.e.,* local species number density fluctuations occurred in the solution. These heterogeneities can be modelled by assuming a length distribution of the effective hard-sphere radius, and because $R_{eHS} >> R_g$, R_{eHS} is independent of the size and geometry of the primary scatterers. Therefore, the length distribution does not affect the form factor of primary species but only their structure factor[11]. This concept is mathematically expressed in Supplementary Equation 4, where the size distribution $D(r)$ is combined with the hard-sphere structure factor, with $<R_{eHS}>$ denoting the intensity averaged effective hard-sphere radius, and σ the corresponding standard deviation and r is an integration variable. The structure factor expressed by Supplementary Equation 4 is further referred to as a polydisperse hard-sphere structure factor, $<S_{HS}(q)>$, and for $D(r)$ we used a Zimm-Schulz-Flory distribution[12]. Supplementary Equation 5 expresses $D(r)$, in which Γ denotes a gamma function, z is related to the width of the distribution, and the standard deviation σ is expressed by Supplementary Equation 6. We present further details about the effect of the hard-sphere structure factor in Supplementary

Note 4, showing an example fit of monodisperse and polydisperse variants of the hard-sphere structure factor.

Supplementary Note 4:

Monodisperse versus polydisperse hard-sphere structure factor

Fitting with the monodisperse $S_{HS}(q)$ structure factor from Supplementary Note 3 and Supplementary Equation 2, introduces oscillations to the fitting curve, which are not present in the original data. This is illustrated by a fit to the pattern collected at 330 seconds based on combining Supplementary Equation 1 (see Supplementary Note 2) and Supplementary Equation 2 (see Supplementary Note 3), where the fit is a blue curve in Supplementary Fig. 3. The red curve in Supplementary Figure 3 shows the improved fit to the experimental data when the expression for the scattering intensity includes a polydisperse $\langle S_{HS}(q) \rangle$ structure factor (Supplementary Note 3, Supplementary Equation 4). For both the monodisperse and polydisperse variants of the hard-sphere structure factor, the curve fittings yielded very similar values of R_{eHS} (monodisperse) and $\langle R_{eHS} \rangle$ (polydisperse) parameters. But, when $\langle S_{HS}(q) \rangle$ was used the size distribution function led to smearing out and dampening of the oscillations (arrows in Supplementary Fig. 3).

Supplementary Note 5: *Mathematical expressions for scattering from the surface fractal aggregates of primary species*

During the first part of stage III between 420 and 840 seconds, in the $q > 1$ nm^{-1} region only negligible variations in shape and intensity were observed (see main text, Figs. 1 and 2B). On the other hand at $q < 1$ nm^{-1} a characteristic increase in intensity occurred, indicating a gradual growth of the larger scattering features.

Beyond 420 seconds, the overall increase in scattering at $q < 1$ nm^{-1} followed a $I(q) \propto q^{-3 > -a > -4}$ (where a is the exponent) dependence indicating that the signal could be attributed to scattering from rough fractal surfaces. Bale and Schmidt[13] and Wong and Bray[14] derived expressions approximating scattering from such features (Supplementary Equation 7). In this expression A is a constant proportional to the surface area of the scattering features (such as crystal surfaces or pore surfaces *etc.*) and Γ denotes a gamma function. Furthermore, the parameter D_s is a surface fractal dimension, where $D_s = 2$ represents smooth surfaces and $D_s \rightarrow 3$ represents very rough fractal surfaces.

As is further evidenced by the detailed analysis of the $\phi V_{part}(\Delta\rho)^2$ and the normalized $\phi(\Delta\rho)^2$ pre-factors for the primary species (Supplementary Note 2), the growth of the surface fractal features proceeded throughout the entire stage III, while $\phi\,(\Delta\rho)^2$ remained constant (main text, Fig. 3B). This indicates, that the growth of the surface fractals can be interpreted as the aggregation of primary species. Thus, Supplementary Equation 7, is as such only applicable to the low-q part of our data, since it implies that $I_{SF}(q \rightarrow \infty) = 0$, and does not allow for the surface fractal to be internally composed of the smaller primary scatterers with form factor $P_{cyl}(q)$. Therefore, Supplementary Equation 7 was re-written as a structure factor $S_{SF}(q)$ to allow the contribution from the smaller structural units. This way, the following necessary conditions can be fulfilled: in the low-q regime $P_{cyl}(q \rightarrow 0) = 1$ and for high-q $S_{SF}(q \rightarrow \infty) = 1$ (Supplementary Equation 8). In Supplementary Equation 8, the $SF(q,D_s)$ part is defined in the same way as in Supplementary Equation 7. However, the difference between the two expressions is in the constants, where A was defined as being proportional to the total surface of all scattering features. In contrast,

A' is proportional to the *relative* surface of all scattering features formed from the aggregating primary species and is normalized against the form factor in order to fulfil the condition that $S_{SF}(q \rightarrow \infty) = 1$. The scattering patterns collected after 420 seconds (see main text, Figs. 1 and 2B), when the intensity starts to increase at low-q, mark the onset of very rapid aggregation of the primary scatterers, which in stage II only loosely interacted forming domains of widely-spaced individual scatterers. This process is dominated at low-q by the scattering from the growing surfaces of these aggregates. It is worth noting that the inter-particle interactions between 420 and 510 seconds were also affected by the minor contribution from $<S_{HS}(q)>$, as we discuss in the Supplementary Note 6 below.

Supplementary Note 6:

Structure factor during the transition between stages II and III

In order to explain the scattering contributions in the patterns collected between 420 and 510 seconds (*i.e.,* the transition between stages II and III), we considered a collection of primary species, in which some of the units would be grouped into domains of scatterers that interact via a hard-sphere structure factor $<S_{HS}(q)>$, but at the same time some of those units would start forming larger, densely-packed aggregates. These new aggregates would be composed, at short length-scales, of primary species, but arranged in such a way that at larger length-scales the structure would appear to be a solid coarse surface. In such a case, the low-q regime of the scattering curves, where by definition $P_{cyl}(q \rightarrow 0) = 1$, could be characterised by the linear combination of scattering from co-existing hard-sphere structures (domains of primary species) and surface fractals (internally denser aggregates of primary species). This is in contrast to the high-q regime which is

dominated by scattering from the form factor and where the structure factor is assumed as $S(q \rightarrow \infty) = 1$. The effective structure factor of such a collection of primary species can be expressed by the *semi-empirical* Supplementary Equation 9. Here, the components of the sum were defined in Supplementary Notes 3 and 5, with A' being proportional to the relative surface of all scattering features and normalized against the form factor in order to fulfil the condition that $S(q \rightarrow \infty) = 1$. Between 420 and 510 seconds both contributions followed the fit as expressed by Supplementary Equation 1, but due to the decreasing local volume fraction v (obtained from the fits), the relative contribution from $\langle S_{HS}(q) \rangle$ became small relatively fast compared to the quickly-evolving surface fractal term. After 510 seconds the scattering model contained exclusively the latter term. Hence, we assumed that after 510 seconds $\langle S_{HS}(q) \rangle \rightarrow 1$, and the expression for the resulting structure factor yielded $S_{SF}(q)$ (Supplementary Equation 8). Supplementary Figure 4 shows a scattering curve at 450 seconds with a fit to the data. For the sake of comparison the respective contributions from $\langle S_{HS}(q) \rangle$ and $S_{SF}(q)$ are also plotted.

Supplementary Note 7:

Structure factor and form factor during the transition between stages III and IV

During stages III and IV (main text, Fig. 1) the increase in intensity is dominated by a change at $q < 1$ nm^{-1}. We modelled these changes in terms of surface fractal contributions using the above described scattering model which fitted well all patterns up to ~1500 seconds. However, as the system further developed, the scattering curves showed considerable changes in their shape at $q > 1$ nm^{-1} indicating the growth of the primary species. As illustrated in Supplementary Figure 5 the scattering curves at 720 and 1500 seconds are fitted with a cylindrical form factor and

a surface fractal structure factor $S_{SF}(q)$. Even though at 3000 seconds the low-q part of the scattering curve could still be fitted by $S_{SF}(q)$, at high-q the form factor changed. Thus, the expression for the cylindrical form factor was no longer valid and the scattering curves for stage IV (between 1500 and 5400 seconds) were fitted only partially with an expression for the scattering intensity including $S_{SF}(q)$ (Supplementary Note 6). The auxiliary cylindrical form factor expression was only used to extract the $\phi V_{part}(\Delta\rho)^2$ pre-factor values (Supplementary Note 2), which enabled the correct scaling of the structure factor. For scattering expressed by a form factor on its own, the $\phi V_{part}(\Delta\rho)^2$ pre-factor corresponds to $I(q=0)$ because $P_{cyl}(q=0) = 1$, which is no longer true when scattering at low-q is modified by a contribution from a structure factor. However, because by definition $S(q \rightarrow \infty) = 1$, we could use an approximate form factor to derive the values for $\phi V_{part}(\Delta\rho)^2$ by simultaneously extrapolating the form factor to low-q and the structure factor to high-q. This way the pre-factor is the minimized parameter that is matching the mutual scaling of both factors (see Supplementary Fig. 5). As a result of this fitting the characteristic parameters that represent growth of surfaces throughout stage IV could be obtained without the form factor.

Supplementary Note 8:

Evolution in the morphology of $CaSO_4$ precipitates after 5400 seconds

In the main text, we showed the development in the SAXS patterns up to 5400 seconds for the case of 50 mmol/L $CaSO_4$ at 12 °C. This period contains the most significant changes in SAXS and is therefore the most relevant part for revealing the mechanisms of $CaSO_4$ nucleation, growth and transformation under the aforementioned physicochemical conditions. Nonetheless, the actual measurements

were performed up to ~4 hours (14370 seconds). In this later period very limited changes were observed in the SAXS signal and therefore only the final scattering pattern is compared to the last scattering pattern from stage IV at 5400 seconds (Supplementary Figure 6). Throughout most of the reaction we dealt with isotropic scattering as indicated by the circular shape of the scattered intensity in 2D (presented in Supplementary Fig. 6A for 5400 seconds). However, during the last stages of $CaSO_4$ growth the 2D SAXS patterns became anisotropic, as shown in Supplementary Figure 6A for 14370 seconds. If the particles are sufficiently large and elongated, they will become aligned with respect to their long-axes in the horizontally mounted capillary of the flow-through cell (Supplementary Fig. 6B). Furthermore, if within the accessible q-range there are any orientation-dependent internal variations in the microstructure of particles with respect to their long-axes, the resulting 2D SAXS pattern would be anisotropic. In Supplementary Figure 6A for the pattern collected at 14370 seconds, stronger scattering at higher angles is observed in the direction almost parallel to the Y axis of the detector (vertical direction), and thus normal to X axis (horizontal direction). Such a scattering pattern is expected from morphologies in which larger dimensions of anisotropic features are oriented along the flow (and long-axes of the particles containing these features, Supplementary Fig. 6B), and smaller features are perpendicular to the flow (and hence perpendicular to long-axes of particles, Supplementary Fig. 6B). In Supplementary Figure 6C we compare 1D scattering curves from the isotropic case at 5400 seconds, with those from the anisotropic case representing scattering contributions from long- and short-axis of anisotropic particles at 14370 seconds. It can be seen that only minor changes in SAXS intensity occurred between 5400 and 14370 seconds, but characteristically at high-q the scattering pattern shifted slightly further towards lower q-values during the

more advanced stage of the process. This shows that beyond 5400 seconds, at the nano-scale, internal building units continued to grow, and larger morphologies were observed for the direction parallel to the flow.

Supplementary Note 9:

Selected physicochemical properties of $CaSO_4{\cdot}xH_2O$ polymorphs

In Supplementary Table 2 we present the relevant physicochemical information regarding the three calcium sulphate polymorphs. The solubility of $CaSO_4{\cdot}xH_2O$ in aqueous solutions was calculated with the geochemical code PHREEQC[15], and is based on bulk solubility data. Based on Supplementary Table 2, we calculated that for a 50 mmol/L $CaSO_4$ solution at 12 °C.

Without taking into account the solubility, one would expect approximate volume fractions, ϕ, of 0.369% - 0.373% for gypsum, 0.263% - 0.270% for bassanite, and 0.229% for anhydrite. By taking into account the bulk solubilities in pure water at 12 °C, the expected ϕ is 0.256% - 0.258% for gypsum and 0.104% for anhydrite, whereas for bassanite no solid phase is expected (*i.e.,* undersaturated). Finally, when the solubility in an actual solution containing the dissolved Na^+ and Cl^- is also considered (which are the counter-ions to the Ca^{2+} in $CaCl_2$ and to SO_4^{2-} in Na_2SO_4 in our original stock solutions; see Methods), the expected ϕ is 0.201% - 0.203% for gypsum and 0.062% for anhydrite, whereas again for bassanite no solid phase is expected.

The calculated volume fraction values do not take into account the change in volume between a solution containing only a fully dissolved phase and one with a solid phase. However, this change is negligible because only low concentrations of reagents are dissolved, and thus, the calculated volume fractions based on the above

data can be regarded as a very close approximation. We used the predicted values of ϕ, to calculate the electron densities of what we termed *pseudo*-phases, *i.e.,* the electron densities of phases with an assumed volume fractions for gypsum, bassanite and anhydrite. For instance, if anhydrite was the phase contributing to the scattering of the primary particles, the corresponding volume fraction value would yield the electron density of anhydrite, and therefore the actual extracted (rather than expected) value is referred to as a *pseudo*-anhydrite phase. In scattering one considers $\Delta\rho=|\rho_{phase} - \rho_{solvent}|$, and when using the electron density of the solvent (water) of 334 e^-/nm^3, we could calculate the density of the newly forming phase, ρ_{phase}. The results are presented in Fig. 4 in the main text.

Supplementary References:

1. Momma K, Izumi F. VESTA 3 for three-dimensional visualization of crystal, volumetric and morphology data. *Journal of Applied Crystallography* **44**, 1272-1276 (2011).

2. Hawthorne F, Ferguson R. Anhydrous sulphates. II : Refinement of the crystal structure of anhydrite. *The Canadian Mineralogist* **13**, 289-292 (1975).

3. Ballirano P, Maras A, Meloni S, Caminiti R. The monoclinic I2 structure of bassanite, calcium sulphate hemihydrate (CaSO4· 0.5 H2O). *European Journal of Mineralogy* **13**, 985-993 (2001).

4. Henry PF, Weller MT, Wilson CC. Neutron powder diffraction in materials with incoherent scattering: an illustration of Rietveld refinement quality from nondeuterated gypsum. *Journal of Applied Crystallography* **42**, 1176-1188 (2009).

5. Van Driessche A, Benning L, Rodriguez-Blanco J, Ossorio M, Bots P, García-Ruiz J. The role and implications of bassanite as a stable precursor phase to gypsum precipitation. *Science* **336**, 69-72 (2012).

6. Van Driessche AE, García-Ruiz JM, Delgado-López JM, Sazaki G. In situ observation of step dynamics on gypsum crystals. *Crystal Growth & Design* **10**, 3909-3916 (2010).

7. Morales J, Astilleros JM, Fernández-Díaz L. Letter. A nanoscopic approach to the kinetics of anhydrite (100) surface growth in the range of temperatures between 60 and 120° C. *American Mineralogist* **97**, 995-998 (2012).

8. Guinier A, Fournet G. *Small angle scattering of X-rays* (1955).

9. Beaucage G. Approximations Leading to a Unified Exponential/Power-Law Approach to Small-Angle Scattering. *Journal of Applied Crystallography* **28**, 717-728 (1995).

10. Kinning DJ, Thomas EL. Hard-sphere interactions between spherical domains in diblock copolymers. *Macromolecules* **17**, 1712-1718 (1984).

11. Besselink R, Stawski TM, Castricum HL, ten Elshof JE. Evolution of microstructure in mixed niobia-hybrid silica thin films from sol–gel precursors. *Journal of Colloid and Interface Science* **404**, 24-35 (2013).

12. Flory PJ. Molecular Size Distribution in Three Dimensional Polymers. III. Tetrafunctional Branching Units. *Journal of the American Chemical Society* **63**, 3096-3100 (1941).

13. Bale HD, Schmidt PW. Small-Angle X-Ray-Scattering Investigation of Submicroscopic Porosity with Fractal Properties. *Physical Review Letters* **53**, 596 (1984).

14. Wong P-z, Bray AJ. Small-angle scattering by rough and fractal surfaces. *Journal of Applied Crystallography* **21**, 786-794 (1988).
